# The Hospital Officer's Bookshelf

**Published:** 1912-09-21

**Authors:** 


### THE HOSPITAL OFFICER'S BOOKSHELF.

Hospital Sisters and their Duties. By Eva C. E. Lückes, Matron of the London Hospital. Fourth Edition. (The Scientific Press, Ltd., 28 and 29 Southampton Street, W.C. 1912. Pp. 234. Price 2s. 6d. net.)

Of books which instruct, or seek to instruct, those who are being trained in hospitals as nurses there is a superabundance. Many are excellently well designed for helping the probationer to understand the why and the wherefore of much that she is taught by the ward sister or staff nurse to do; and the best of them are indispensable. Yet they all stop short with the conclusion of the probationary training. Of post-graduate text-books, so to speak, there is as great a dearth as there is a glut of the other sort. Miss Lückes perceived the need and the omission, and set herself to remedy it. The success of her novel venture is sufficiently attested by the fact that it has now reached a fourth edition. This, as it happens, has been so largely recast and rewritten that it has the freshness and in large part the merit of an entirely new book. In successive chapters Miss Lückes deals with the various departments which fall to the lot of a ward sister to supervise and control. The ability to teach, to direct, to command is one which no book can impart; it is a quality of the individual which either does or does not exist; when once the character has been "formed" by heredity, by environment, by growth and circumstance, it can neither be gained nor lost. This power it is essential for a successful sister to possess; and although, as has been said, it is not a thing which can be learnt from books, at least Miss Lückes draws us a picture of the woman who has it and of the way in which it should be used in hospital. The ideal sister is set forth in these pages fully and sympathetically; if she existed in life we feel that we should recognise her from the description. The fact that such a person does not, and cannot, exist is no detraction from the skill with which her portrait is drawn. Whether Miss Lückes' success has deterred others from competing with her we do not know; but certainly if there is to be only one book on the duties of ward sisters it is fortunate that it should be this. Our only suggestion for its improvement is the omission of various not over-relevant quotations, and of occasional lapses into a somewhat mawkish sentimentality.

Statistics of Puerperal Fever and Allied Infectious Diseases. By George Geddes, M.D. (Bristol : John Wright and Sons, Ltd. 1912. Pp. 119. Price 6s. net.)

Dr. Geddes holds the view that puerperal fever is "largely if not entirely due to contamination, directly by, or indirectly through, suppurating wounds. . . ." In support of this thesis he has collected an enormous number of statistics concerning death rates, accidents, populations, and other features of a great number of districts, many of them in Lancashire; and these complicated statistics occupy a large part of his book. He further holds that the responsibility for puerperal sepsis rests more often on the doctors than with midwives. Dr. Geddes certainly brings enthusiasm and energy to his task; but he speaks as if suppurating wounds due to industrial accidents were the sole causes of septic infection. If the medical men of Lancashire are in the habit of soaking their hands daily in pus and then without antiseptic precautions attending midwifery cases, there may be something in his argument; but surely this cannot be the case. Gloves Dr. Geddes seems never to have heard of in midwifery as part of the prophylaxis of puerperal sepsis. We do not anticipate that he will convince the profession that his statistics mean all that he thinks they do, or that his book is worth the comparatively high price charged for it.

